# Correction: Molecular bowls for inclusion complexation of toxic anticancer drug methotrexate

**DOI:** 10.1039/d5sc90250a

**Published:** 2025-11-07

**Authors:** Pratik Karmakar, Tyler J. Finnegan, Darian C. Rostam, Sagarika Taneja, Sefa Uçar, Alexandar L. Hansen, Curtis E. Moore, Christopher M. Hadad, Kornkanya Pratumyot, Jon R. Parquette, Jovica D. Badjić

**Affiliations:** a Department of Chemistry and Biochemistry, The Ohio State University 100 West 18th Avenue Columbus Ohio 43210 USA badjic.1@osu.edu; b Supramolecular Chemistry Research Unit, Department of Chemistry, Faculty of Science, King Mongkut’s University of Technology Thonburi 126 Pracha Uthit Road, Bang Mod, Thung Khru Bangkok 10140 Thailand; c Atatürk University, Faculty of Science, Department of Chemistry Erzurum 25240 Turkey; d Campus Chemical Instrumentation Center, The Ohio State University 100 West 18th Avenue Columbus Ohio 43210 USA

## Abstract

Correction for ‘Molecular bowls for inclusion complexation of toxic anticancer drug methotrexate’ by Pratik Karmakar *et al.*, *Chem. Sci.*, 2024, **15**, 10155–10163, https://doi.org/10.1039/D3SC05627A.

The authors regret the omission of a funding acknowledgement in the original article. The corrected acknowledgement is given here.

The Royal Society of Chemistry apologises for these errors and any consequent inconvenience to authors and readers.

